# Comment on “Precision leak detection in the golden window: emerging biomarker strategies for anastomotic integrity monitoring after low anterior resection”

**DOI:** 10.1097/JS9.0000000000003054

**Published:** 2025-07-14

**Authors:** Hui Liu, Fengting Zhu

**Affiliations:** a Department of Interventional Radiology, Haikou Affiliated Hospital of Central South University Xiangya School of Medicine, Haikou, Hainan Province, China; b Department of Oncology, Guangdong Provincial Hospital of Traditional Chinese Medicine Hainan Hospital, Haikou, Hainan Province, China

*Dear Editor,*

We read with great interest the recent review titled “Precision Leak Detection in the Golden Window: Emerging Biomarker Strategies for Anastomotic Integrity Monitoring After Low Anterior Resection^[1]^.” The article provides a timely and comprehensive synthesis of current biomarker strategies across different biological phases of healing. However, we would like to highlight an important methodological consideration that warrants further clarification or future investigation. This manuscript was prepared in accordance with the TITAN Guidelines 2025 for governing declaration and use of artificial intelligence in scholarly publishing^[2]^. No AI tools were used in this work.

While the review effectively categorizes and discusses multiple biomarkers, it overlooks several important perioperative variables that are known to influence the risk of anastomotic leakage (AL) and may confound the interpretation of biomarker expression. For instance, the use of transanal drainage tubes and protective ileostomies has been associated with changes in leak incidence and local inflammatory responses^[3,4]^. Moreover, factors such as tumor location and the administration of neoadjuvant radiotherapy can significantly impact anastomotic healing capacity and consequently affect the likelihood of leakage^[5,6]^.

Omission of these variables may limit the generalizability of the biomarker findings across surgical contexts. We suggest that future systematic analyses incorporate these clinical factors as potential stratification variables or covariates in pooled evaluations. Doing so would enhance the translational utility of the proposed diagnostic strategies. We commend the authors for their valuable work and hope our comments will contribute constructively to ongoing efforts in improving early AL detection.

## Data Availability

The letter does not have any data.

## Research registration unique identifying number (UIN)

Not registered for the type of the study.
